# Lipid metabolism and inflammation modulated by Vitamin D in liver of diabetic rats

**DOI:** 10.1186/s12944-015-0030-5

**Published:** 2015-04-18

**Authors:** Conghua Ning, Lina Liu, Guodong Lv, Ye Yang, Yuanyuan Zhang, Rui Yu, Yongtao Wang, Jun Zhu

**Affiliations:** Department of Endocrinolog, First Affiliated Hospital of Xinjiang Medical University, No. 137 Liyushannan Road, Urumqi, 830054 Xinjiang China; Institute of Research, The First Affiliated Hospital of Xinjiang Medical University, Urumqi, 830054 Xinjiang China

**Keywords:** Diabetes-induced liver injury, Vitamin D, NF-κ B, PPAR-α

## Abstract

**Background:**

In recent years, much evidence suggested that vitamin D plays an important role in decreasing the risk of type 2 diabetes. The purpose of this study was to investigate whether 1, 25 (OH) _2_D_3_ can modulate inflammation and lipid metabolism in type 2 diabetic rat liver.

**Methods:**

Type 2 diabetes was induced in SD rat with high-fat and high-sugar diets and multiple low-dose streptozotocin. The levels of serum calcium, phosphorus, glucose, TC, TG, AST, ALT and hepatic TG were determined. H & E staining were performed to assess the effects of vitamin D treatment on pathological changes in the liver tissues. Immunohistology, real-time PCR and Western blot were used to evaluate the expressions of NF-κ B, MCP-1, ICAM-1, TGF-β1, PPAR-α and CPT-1.

**Results:**

The administration of 1, 25 (OH) _2_D_3_ reduced liver weight. Compared to DM rats, 1, 25 (OH) _2_D_3_-treated DM rats had lower liver weight. Moreover, compared to healthy or 1, 25 (OH) _2_D_3_-treated DM rats, DM rats had increased hepatic transcription factors (NF-κ B), monocyte chemoattractant protein −1 (MCP-1), intercellular adhesion molecule −1 (ICAM-1), transforming growth factor-β1 (TGF-β1) expressions, but had fewer hepatic PPAR- α and CPT-1 expressions.

**Conclusions:**

1, 25 (OH) _2_D_3_ significantly modulated the liver inflammation and lipid metabolism in diabetic rat models, which may be caused by its regulations on hepatic signaling NF-κ B pathway and PPAR- α.

## Introduction

Type 2 diabetes mellitus (T2DM) is one of the main noncommunicable chronic diseases with a growing prevalence; it affected 382 million people worldwide in the year of 2013 and is expected to affect 592 million by 2035. Chronic complications of diabetes, which involve coronary artery disease, renal, liver and ophthalmologic diseases, is the primary cause of disability and mortality in diabetes mellitus (DM) patients. It is reported that the standardized mortality rate from end-stage liver disease (i.e. cirrhosis) is higher than that for cardiovascular disease among patients with diabetes [1,2]. Liver disease is one of diabetic complications, and should be well addressed [3].

Vitamin D is a fat-soluble vitamin, which is an essential micronutrient with major implications for human health [4]. The biologically active form of vitamin D is 1, 25 (OH) _2_D_3_ (also known as active vitamin D3 [5]. Vitamin D receptors are widely distributed in more than 38 tissues [6]. Macrophages and dendrite cells constitutively express Vitamin D receptors, which indicates vitamin D plays an important role in regulating the inflammatory response [7,8]. Several studies have confirmed the involvement of vitamin D in modulating the inflammatory response [9,10]. In recent years, much evidence suggested that vitamin D plays an important role in decreasing the risk of many chronic diseases, including type 2 diabetes [11], the metabolic syndrome [12] and cardiovascular disease [13].

Diabetes-induced liver injury includes inflammatory response, lipid accumulation and liver fibrosis [14]. T2DM produces a state of chronic hyperglycemia and insulin resistance, which leads to the increasing of intracellular reactive oxygen species (ROS) levels. The accumulated ROS can activate the NF-κ B pathway, leading to the occurrence of liver inflammation [15]. Previous study demonstrated that vitamin D reduces the nuclear translocation of NF-κ B by up-regulating the inhibitor of NF-κ B (I κ B-α) in LPS-stimulated murine macrophages [16]. However, the regulatory effect of 1, 25 (OH) _2_D_3_ on NF-κ B and its downstream inflammatory cytokines expressions in DM hepatocyte remains unclear. In addition, the accumulation of triglycerides within hepatic cell is the mainly characteristic of fatty liver with type 2 diabetes [17]. Peroxisome proliferator-activated receptor α (PPAR-α), which is mainly expressed in the liver, plays a pivotal role in the regulation hepatic lipid metabolism [18]. Motiwala [19] and Zittermann [20] have shown that vitamin D modulates lipid metabolism by decreasing the level of serum triglyceride (TG) in overweight subjects. A recent study have found that 1, 25 (OH) _2_D_3_ modulates cardiac lipid metabolism by affecting the expression of PPAR-α in a DM rat model [21]. It has not yet been studied whether vitamin D can modulate hepatic lipid metabolism by regulating PPAR-α in type 2 diabetic rat liver.

Increasing evidence suggests that the circulating concentration of 25-OH vitamin D was negatively associated with the risk of liver disease [22,23]. However, given that the regulatory mechanisms of 1, 25 (OH) _2_D_3_ on liver have not been completely elucidated, the purpose of this study was to investigate whether 1, 25 (OH) _2_D_3_ can modulate inflammation and lipid metabolism in type 2 diabetic rat liver.

## Materials and methods

### Animal modeling and grouping

The experimental designs and protocols for animal studies were reviewed and approved by Xinjiang management committee for medical laboratory animal sciences. A total of 50 male SD rats were provided by the Xinjiang Disease Control and Prevention Center. Rats were housed in standard cages and maintained on rat chow and tap water ad libitum. All rats were housed for 1 week prior to diet intervention. We randomly selected 15 rats as normal control group (NC group), which were fed with normal diet. The remaining rats were used to establish DM models. They were fed with high-fat and high-sugar diets, containing 10% refiing lard, 20% sucrose, 2% cholesterol, 8% custard powder, and 60% of normal diet, for indicated duration (eight weeks). Then these rats were subjected to the intraperitoneal injection of 35 mg/kg streptozotocin (STZ; Sigma, St. Louis, MO, USA). One week later, the fasting plasma glucose (FPG) and 2-h plasma glucose (2hPG) were measured with a complete blood glucose monitor (ACCU-CHEK, Germany), rats with FPG ≥ 7.0 mmol/L and/or 2hPG ≥ 11.1 mmol/L were considered as DM models.

These DM model rats were randomly divided into diabetic group (DM group) and vitamin D treatment group (VD group, n = 15 per group). Rats of vitamin D treatment group were administrated vitamin D (0.03 μg/kg body weight; Shanghai Roche Pharmaceuticals, Shanghai, China) in 0.05 mL peanut oil once daily via gavage, and equivalent peanut oil was administrated in diabetic group and normal control group for 8 weeks. At the end of the vitamin D intervention period, blood samples were drawn from the abdominal aorta and the serum samples were separated by centrifuge. The entire liver was dissected out, weighed, the tissues were cut into small pieces, immersed in RNA later solution at room temperature, and then stored at −80°C. Remaining liver tissues were fixed in 4% paraformaldehyde.

### Biochemical analysis

Serum calcium (Ca), phosphorus (P), glucose total cholesterol (TC), TG, Alanine aminotransferase (ALT), aspartate aminotransferase (AST), were determined with an automatic biochemistry analyzer (Hitachi 7600, Tokyo, Japan). Hepatic TG contents were determined enzymatically by using commercial kits (Bio Sino Bio-technology and Science Inc, Beijing, China).

### Histopathological staining

The fresh liver tissue was washed with saline, and then fixed in 10% buffered formalin. After dehydrated, these tissues were embedded with paraffin, and then cut into 3 μm sections on a microtome (Leica, Nussloch, Germany) and then stained with H & E.

### Immunohistochemistry

Paraffin sections were de-waxed and re-hydrated through a graded alcohol series. The endogenous peroxidase was removed, and the sections were exposed to antigen retrieval. Then, slides were incubated, respectively, with Rabbit anti-rat MCP-1 (Boster Biological Technology, Wuhan, Hubei, China), rabbit anti-rat ICAM-1 (Boster Biological Technology, Wuhan, Hubei, China), rabbit anti-rat TGF-β1 (Boster Biological Technology, Wuhan, Hubei, China), and rabbit anti-rat CPT-1a (Bioss Biotechnology, Beijing, China) (final concentrations 1:100, 1:100, 1:200, 1:100, respectively) for over-night at 4°C. Then the sections were then incubated with secondary antibodies. After stained with DAB chromogenic reagent (ZSGB-BIO, Beijing, China) and counterstained with hematoxylin, these sections were sealed and then visualized under microscopy with the CM-2000B biomedicine image analysis system (Beihang, Beijing, China). Brown staining was considered as positive. Five fields were randomly selected under high magnification (×400), and the averaged number of positive cells were counted and calculated.

#### Quantitative real-time polymerase chain reaction

Total RNA was extracted from liver tissue (100 mg) with RNeasy Mini kits (Qiagen, Hilden, Germany). QuantiTect Rev Transcription Kits (Qiagen) were used to reverse-transcribed to cDNA, according to manufactures’ instructions. The real-time quantitative PCR assays were performed with QuantiFast SYBR green PCR Master Mix containing ROX as a passive reference (Qiagen), on Bio-Rad iQ5 system (Bio-Rad, Hercules, CA, USA). Primer sequences used were listed in Table 1. Quantitative PCR amplification conditions were as follows: melt at 95°C for 5 min; and at 95°C for 50 s, 60°C for 30 s, for 40 cycles. The housekeeping gene b-actin was used as a reference gene for normalization. Primers used for rat genes were as following: NF-κ B p65: F: 5′-TCA CGG GAC CTG GCT GGG AG-3′,R: 5′-CCG CCG AAG CTG CAT GGA CA-3′;MCP-1:F: 5′-CAG CCA GAT GCA GTT AAT GCC-3′,R: 5′-AGC CGA CTC ATT GGG ATC AT-3′;ICAM-1:F: 5′-CGT GGC GTC CAT TTA CAC CT-3′,R: 5′-TTA GGG CCT CCT CCT GAG C-3′; TGF-β1:F: 5′-AGA AGT CAC CCG CGT GCT AAT-3′,R: 5′-CAC TGC TTC CCG AAT GTC TGA-3′; PPAR-α: F: 5′-CCTGCCTTCCCTGTGAACT-3′,R:5′-ATCTGCTTCAAGTGGGGAGA-3′;CPT-1:F:5′-GCCAGACGAAGAAC ATTG-3′,R: 5′-CCTTGACCATAGCCA TCC-3′; β- actin: F: 5′-AGT ACC CCA TTG AAC ACG GC-3′,R: 5′-TTT TCA CGG TTG GCC TTA GG-3′. For each gene, relative change in steady state Mrna in samples was determined using the ΔΔCt method, corrected for the housekeeper.

**Table 1:** **Effects of 1,25(OH)2D3 on body and liver weight**

**Group**	**Weight(g)**	**Liver weight (g)**	**Liver⁄Body**
NC	461.71 ± 35	13.20 ± 1.2	2.86 ± 0.22
DM	346.14 ± 14*	24.37 ± 1.5*	7.05 ± 0.44*
VD	362.87 ± 16*	21.57 ± 1.6*#	5.96 ± 0.53*#

NC, normal control group; DM, diabetic group; VD, vitamin D treatment group.

Values are mean ± SD, n = 15 per group.

*P < 0 .05 vs. NC; #P < 0 .05 vs. DM.

### Western blotting

Liver tissue was homogenized in RIPA Lysis Buffer containing PMSF (a protease inhibitor), and Proteins were collected by centrifuging at 12,000 turn at 4°C in centrifuge for 10 min. The protein concentration was measured by Bradford assay. About 50 micrograms of protein were loaded on 5–10% gradient gels. Proteins were transferred to a PVDF membrane (Thermo Scientific) for 120 min at 80 V. The membranes were incubated in blocking buffer for 2 hours. Then they were incubated with different primary antibodies overnight at 4°C, washed with TBST and incubated with secondary antibody for 2 h at room temperature. Antigen antibody complexes were then visualized using Western Breeze (invitrogen, USA).The primary antibodies used here include NF- k B p65(1:1000, Cell Signaling Technology) and PPAR-α(1:1000, Cell Signaling Technology). The intensity of protein bands were quantified using the Quantity One software (Bio-Rad, USA).

### Statistical analysis

All quantitative data are expressed as the mean ± the standard error of the mean (SEM). SPSS 17.0 software was used for statistical analysis. Comparisons between two groups were analyzed via t-test, and comparisons between more than two groups were analyzed via one-way ANOVA to identify differences among means. Pearson correlations were adopted to note the correlation. A value of P < 0.05 was considered statistically significant.

## Result

### Effect of 1,25(OH)2D3 and diabetes on body and liver weight

About the final weight, rats of DM and VD group showed a lower body weight compared to rats of NC group (P < 0.05), whereas the body weight was similar between the DM and VD group (P > 0.05, Table 1).

The liver weight was measured and showed a significant increase in DM rats than those in NC rats (*P* < 0.05) , the ratio of liver to body weight was still significantly higher. The administration of 1,25 (OH) _2_D_3_ led to a significant reduction in both liver weight and the ratio of liver to body weigh 1,25 (OH) _2_D_3_-treated DM rats compared to DM rats (*P* < 0.05, Table 1).

### Effect of 1,25 (OH) _2_D_3_ on serum metabolic parameters and hepatic TG

There was no significant difference in serum calcium and phosphorus levels of all groups (P > 0.05). Serum glucose level in DM and VD groups was significantly higher than that in NC group (*P* < 0.05). Rats in the DM group exhibited impaired metabolic function as shown by increased serum triglyceride, TC. In the VD group, 1, 25 (OH) _2_D_3_ reduced the level of serum TG, but not serum TC. Our data showed that serum ALT and AST concentrations were extremely elevated in rats of DM group. In the VD group, 1, 25 (OH) _2_D_3_ treatment significantly lowered serum ALT and AST concentrations compared with the DM group. The level of hepatic TG in the DM group was significantly higher than that in NC group (*P* < 0.05), In the VD group, 1, 25 (OH) _2_D_3_ reduced the level of hepatic TG (Table 2).

**Table 2:** **Effects of 1,25(OH)2D3 on serum metabolic parameters and hepatic TG**

**Group**	**Ca**	**P**	**Glucose**	**TC**	**TG**	**ALT**	**AST**	**Hepatic TG**
	**(mmol/L)**	**(mmol/L)**	**(mmol/L)**	**(mmol/L)**	**(mmol/L)**	**(U/L)**	**(U/L)**	**(mg/g)**
NC	2.57 ± 0.10	1.52 ± 0.11	5.61 ± 0.62	1.11 ± 0.09	0.86 ± 0.11	43.09 ± 7.6	69.86 ± 9.9	8.35 ± 1.34
DM	2.61 ± 0.13	1.55 ± 0.08	26.52 ± 3.62*	1.25 ± 0.14*	1.30 ± 0.09*	121.87 ± 14*	150.16 ± 12*	67.74 ± 8.89*
VD	2.63 ± 0.17	1.53 ± 0.08	26.09 ± 1.92*	1.29 ± 0.11*	1.11 ± 0.13*#	95.99 ± 16. *#	105.18 ± 15*#	31.72 ± 7.49*#

Ca, serum calcium; P, serum phosphorus; ALT, alanine aminotransferase; AST, aspartate aminotransferase; NC, normal control group; DM, diabetic group; VD, vitamin D treatment group.

Values are mean ± SD, n = 15 per group.

*P < 0 .05 vs. NC; # P < 0 .05 vs. DM.

### 1, 25 (OH) _2_D_3_ attenuated diabetes-induced hepatic damage

**Figure 1:**
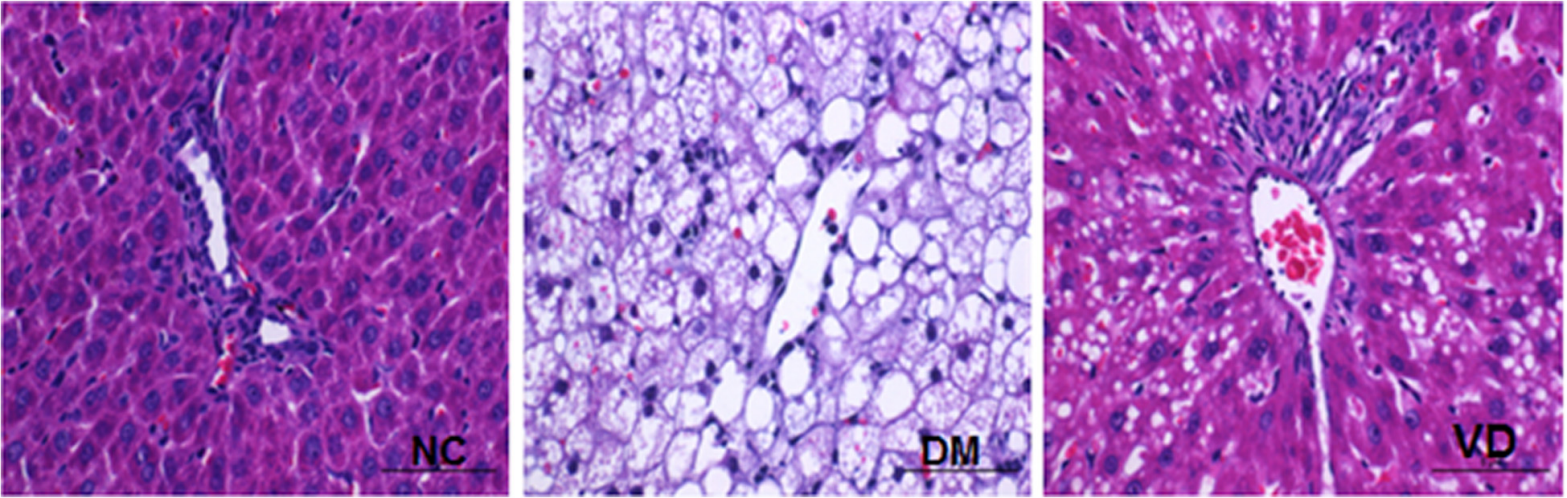
Effects of 1,25 (OH) _2_D_3_ on liver histology. Histological analysis of steatosis in liver sections stained with H & E (magnification 400 ×). NC, normal control group; DM, diabetic group; VD, vitamin D treatment group.

Liver pathology with H & E staining is presented in Figure 1. The hepatic cell structure in normal control group was normal and clear without inflammation and necrosis. In the liver of DM group, diabetes increased hepatic damage, and the morphological change was obviously necrotic and/or inflammatory foci. However, in the liver of 1, 25 (OH) _2_D_3_-treated DM group, the morphological change was less severe with less inflammatory and/or necrotic foci as compared to the liver of Diabetes group.

### Increased NF-κ B and its downstream inflammatory cytokines expression in liver tissues from the DM group and suppressed by 1, 25 (OH) _2_D_3_

**Figure 2:**
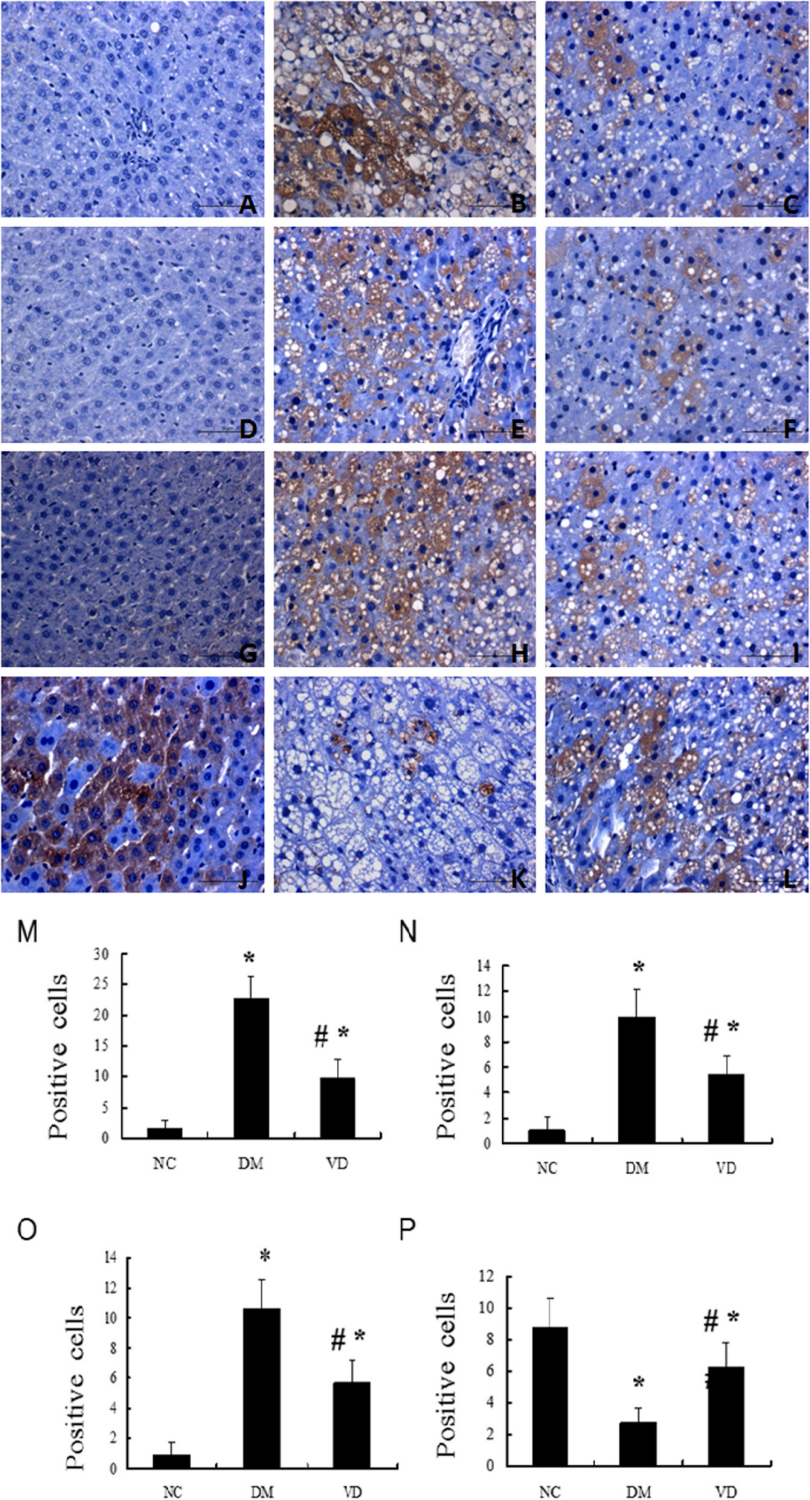
Immunohistochemistry (IHC) staining for MCP-1, ICAM-1, TGF-β1, CPT-1. IHC staining for MCP-1 (**A-C**; ×400); IHC staining for ICAM-1 (**D-F**; ×400); IHC staining for TGF-β1 (**G-I**; ×400), IHC staining for CPT-1 (**J-L**; ×400). The averaged number of positive cells of MCP-1 **(M)**, ICAM-1 **(N)**, TGF-β1 **(O)**, CPT-1 **(P)**. NC, normal control group; DM, diabetic group; VD, vitamin D treatment group. *P < 0 .05 vs. NC; #P < 0 .05 vs. DM.

**Figure 3:**
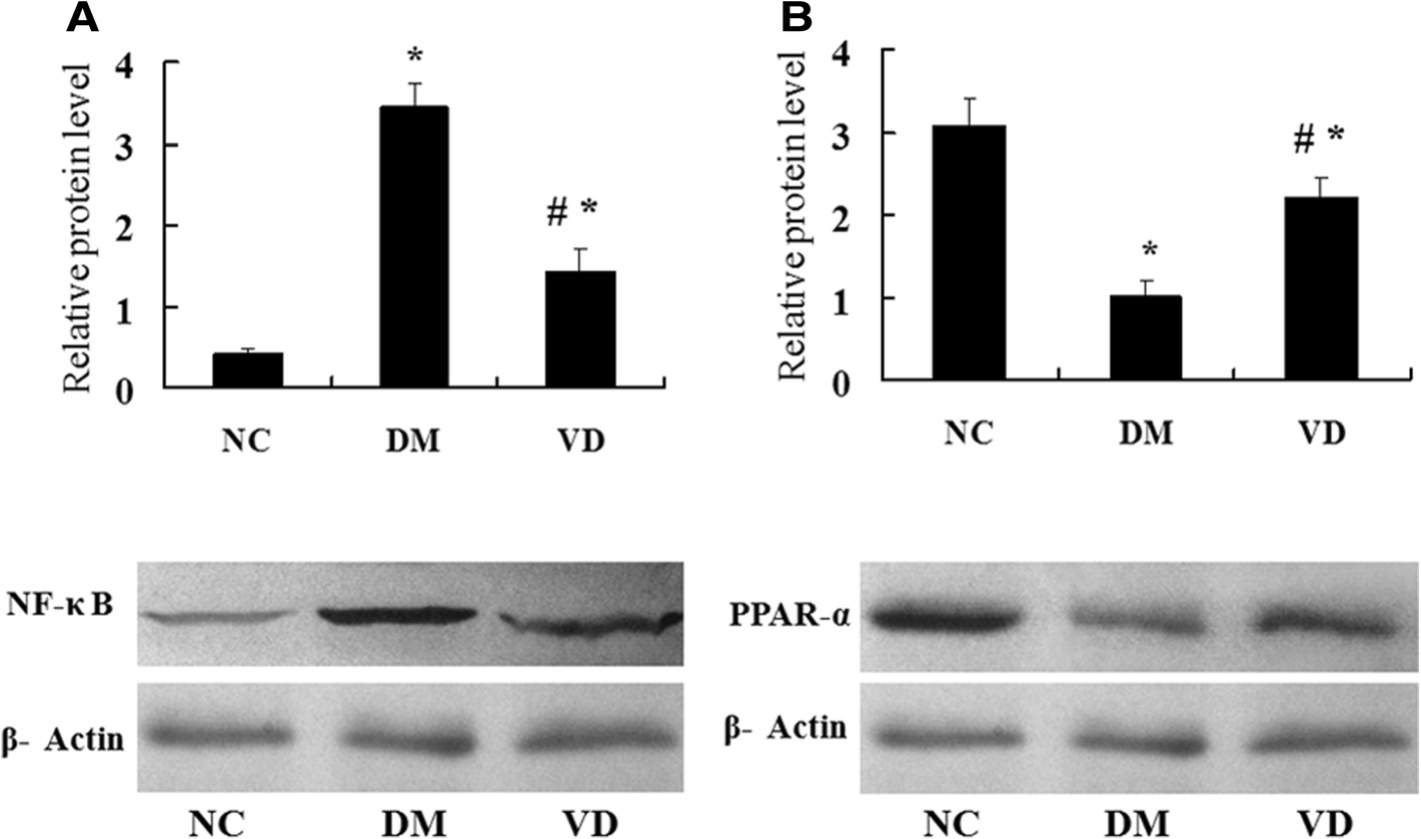
Western blot analysis of protein expression NF-κ B and PPAR-α. Representative western blot images and quantitative analysis of NF-κ B **(A)**, PPAR-α **(B)**. β-actin was used as a loading control. NC, normal control group; DM, diabetic group; VD, vitamin D treatment group. *P < 0 .05 vs. NC; #P < 0 .05 vs. DM.

**Figure 4:**
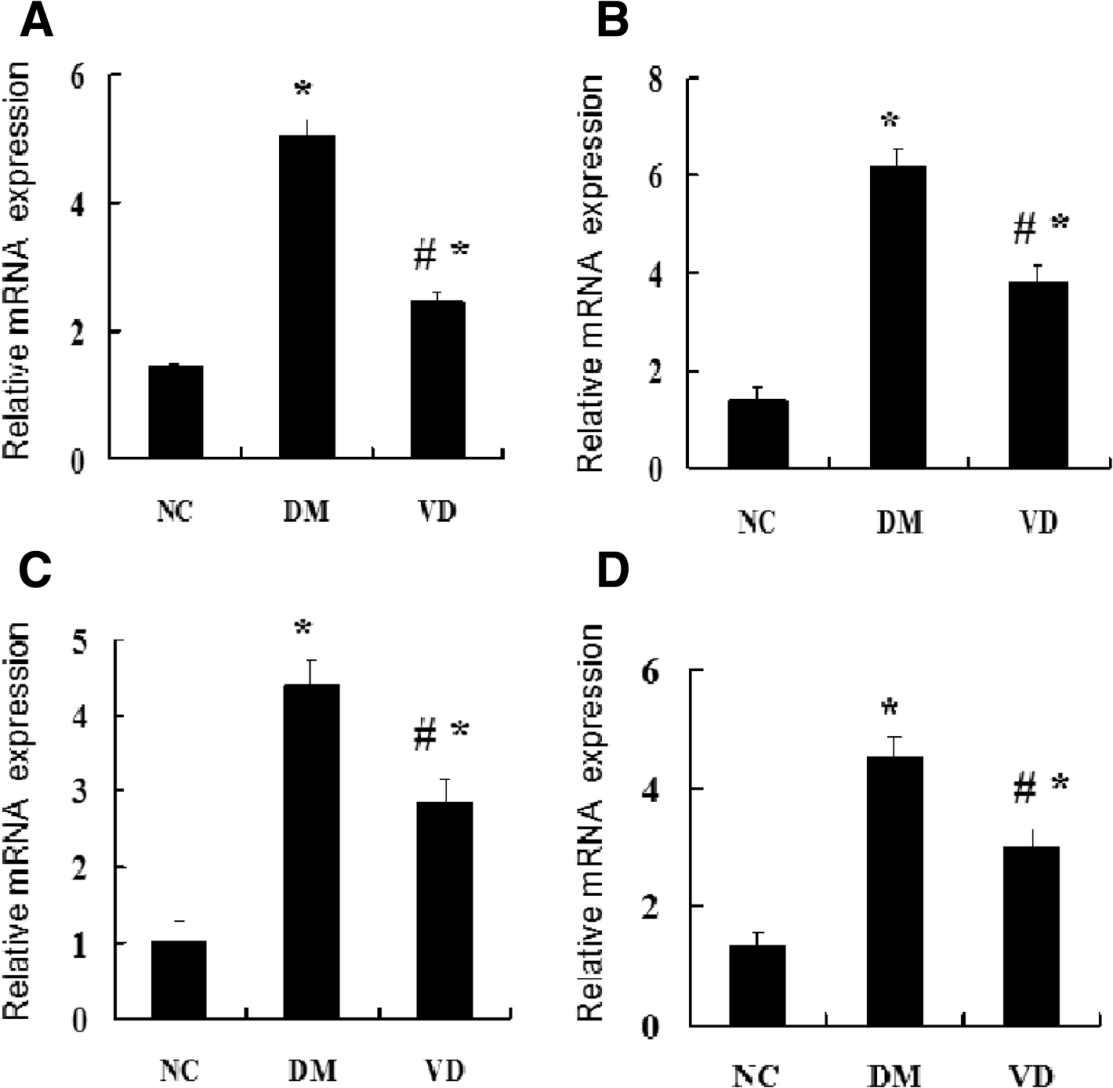
Effects of 1,25 (OH) _2_D_3_ on the expression of hepatic NF-κ B and the related genes. The mRNA expression of hepatic NF-κ B and related genes were assessed. The relative mRNA levels were determined by quantitative RT-PCR and normalized to β-actin. Each gene expression was determined by the 2-ΔΔCt method. **(A)** The mRNA level of hepatic NF-κ B. **(B)** The mRNA level of MCP-1, **(C)** The mRNA level of hepatic ICAM-1, **(D)** The mRNA level of hepatic TGF-β1. Data are expressed as mean ± SD of each group (n = 15 per group). NC, normal control group; DM, diabetic group; VD, vitamin D treatment group. *P < 0 .05 vs. NC; #P < 0 .05 vs. DM.

To test whether hepatic NF-κ B signaling pathway involves in the mechanism of diabetes-induced hepatic damage, we examined their expression by different methods. IHC staining for MCP-1, ICAM-1, and TGF-β1 in livers from the DM group were both strongly positive, whereas they were almost negative in the normal control group , the amount of brown staining granules and the covered area were significantly decreased in the 1, 25 (OH) _2_D_3_-treated DM group, compared with the DM group (Figure 2). Western blot analysis showed that hepatic NF-κ B protein levels in the DM group were significantly increased compared with those of the normal control group; 1, 25 (OH) _2_D_3_ treatment significantly reduced NF-κ B protein levels compared with DM group (Figure 3A), as was hepatic NF-κ B, MCP-1, ICAM-1, and TGF-β1 mRNA (Figure 4). 1, 25 (OH) _2_D_3_ administration suppressed the expression of NF-κ B, MCP-1, ICAM-1, and TGF-β1. Hepatic NF-κ B, MCP-1, ICAM-1, and TGF-β1 mRNA levels of the 1,25 (OH) _2_D_3_treated DM group were all strongly reduced than those from the DM group (Figure 4), as was hepatic NF-κ B protein levels (Figure 3A).

### 1,25 (OH) _2_D_3_ up-regulated the PPAR-α and CPT-1 expression, which involved in lipid oxidation

**Figure 5:**
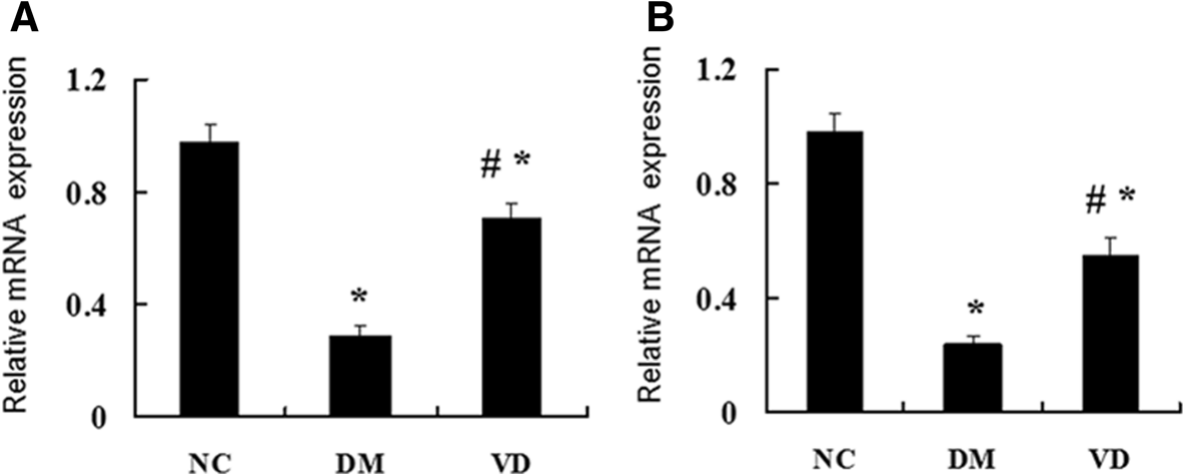
Effects of 1,25 (OH) _2_D_3_ on the expression of hepatic PPAR-α and the related genes CPT-1. The mRNA expression of hepatic PPAR-α and related genes were assessed. The relative mRNA levels were determined by quantitative RT-PCR and normalized to β-actin. Each gene expression was determined by the 2-ΔΔCt method. **(A)** The mRNA level of hepatic PPAR-α. **(B)** The mRNA level of CPT-1. Data are expressed as mean ± SD of each group (n = 15 per group). NC, normal control group; DM, diabetic group; VD, vitamin D treatment group. *P < 0 .05 vs. NC; #P < 0 .05 vs. DM.

The cytokines involved in the regulation of hepatic lipid b-oxidation were assessed by different methods. IHC staining for PPAR-α and CPT-1 in livers from the normal control group were both strongly positive, whereas the amount of brown staining granules and the covered area were significantly decreased in the DM group. 1, 25 (OH) _2_D_3_ treatment significantly increased the amount of brown staining granules and the covered area compared with DM group (Figure 2). Western blot analysis showed that a drastic decrease in hepatic protein level of PPAR-α was found in the DM group when compared to that of normal control group, 1, 25 (OH) _2_D_3_ treatment reversed the expression of PPAR-α (Figure 3B), as was hepatic PPAR-α and CPT-1 mRNA, 1, 25 (OH) _2_D_3_ administration up-regulated the expression of PPAR-α and CPT-1 in liver (Figure 5).

## Discussion

In the present study, we have demonstrated that 1, 25 (OH) _2_D_3_ has protective effects on livers of diabetic rat by modulating inflammation and lipid metabolism. Moreover, the data reported here suggest that 1, 25 (OH) _2_D_3_ down-regulates the expression of the NF-κ B signaling pathway and up-regulates the expression of PPAR-α to attenuate the diabetes-induced liver injury.

Chronic inflammation is involved in the development of hepatic disease [24,25].With the increasing severity of liver disease, the expression of hepatic pro-inflammatory cytokines also increased [26]. The transcription factor NF-κ B and related mechanisms have gradually become a new hot spot in related research field. Multiple studies have demonstrated that the transcription factor NF-κ B play a crucial role in the decision between life and death of a hepatocyte [27]. It is the “genetic switch” of inflammation chain and regulates the gene expression including cellular proliferation, the inflammatory response, chemokines and cell adhesion molecules [28]. Cai D [29] reported that the activity of NF-κ B in the diabetic liver was significantly higher. MCP-1, which is produced mainly by macrophages and endothelial cells, is a potent chemotactic factor for monocytes [30]. It is considered that MCP-1 was specifically activated by NF-κ B in the presence of high glucose [31]. Several studies have reported a correlation between blood and hepatic levels of MCP-1 and the extent of inflammation both in human and animals [32,33]. Moreover, it is suggested that MCP-1 contributes to liver fibrosis, independent of effects on steatosis and inflammation [34]. ICAM-1 is a cytokine responsive integral membrane receptor expressing on the vascular endothelium, and it has been known to play an important role in the development of diabetic pathology [35]. HG-induced up-regulation of NF-κ B promotes ICAM-1 expression through PKC and mitogen-activated protein kinase (MAPK) pathways [36]. TGF-β1 is a multifunctional cytokine, it participates in inflammatory processes, induces fibrosis, causes the suppression of immune response [37,38]. Liver cells can produce TGF-β1, and it plays an important role in inflammation in the liver [39]. It was also suggested that TGF-β1 is a potent profibrogenic factor secreted by activated HSCs, which contributes to the extra cellular matrix (ECM) expression and liver fibrosis [40]. Our results showed that the expression of intra-hepatic NF-κ B and its downstream inflammatory cytokines MCP-1, ICAM-1, and TGF-β1 in DM group are significantly higher than the normal control group, indicating that the liver injury in diabetic rats may be partly associated with the NF-κ B signaling pathway. Emerging data have demonstrated that vitamin D has anti-inflammatory and antioxidative effects in streptozotocin-induced diabetes [41]. In this study, the effectiveness of 1, 25 (OH) _2_D_3_ in attenuating the increasing of NF-κ B, MCP-1, ICAM-1, and TGF-β1 in liver of DM rats suggests that 1,25 (OH) _2_D_3_ might serve as an anti-inflammatory agent for DM liver disease.

On the other hand, hepatic steatosis is one of the mechanisms of liver injury in diabetes. Previous investigation showed that an impaired fatty acid oxidation can promote the development of hepatic steatosis [18]. Hepatic lipid metabolism is mainly regulated by several nuclear receptors and transcription factors. Peroxisome proliferator-activated receptor a (PPAR-α) is one of the major regulators in fatty acid oxidation, prominently expressed in liver, heart and skeletal muscle, which plays an important role in regulation of genes that are involved in lipids utilization and storage [42]. CPT-1 is a rate-limiting enzyme of mitochondria mediated fatty acids β-oxidation, which can facilitate the first step of long-chain fatty acids entering into mitochondria. Further, CPT-1 was declined in NAFLD state and its gene expression is under the regulation of PPAR-α [43,44]. It is reported that PPAR-α-defective mice exhibit macrovesicular steatosis because of failing to induce fatty acid oxidation in liver [45]. The expression of hepatic PPAR-a was significantly decreased in the state of NAFLD [43]. PPAR-α agonists was shown to prevent the development of steatosis by promoting fatty acid oxidation [46]. To explore whether the protective effects of 1, 25 (OH) _2_D_3_ was related to PPAR a activation, the expression of PPAR-α and its target gene was assessed. Our results showed that the expression of PPAR- α and CPT-1 was significantly declined in DM group, whereas 1, 25 (OH) _2_D_3_ increased the PPAR-α and CPT-1 expression. Up-regulated PPAR-α by 1, 25 (OH) _2_D_3_ in liver may promote β-oxidation and results in decreased TG levels. CPT-1 was benefit for FFA transport into mitochondrial for further oxidation and thus decreased lipid deposition. Our study only examined lipid profiles in serum but not in liver tissues, because elevated serum cholesterol and triglyceride concentrations are closely associated with hyperglycemia and obesity. Our data suggested that the level of serum TG decreased with the increasing of PPAR-α and CPT-1 expression. These results indicated that PPAR-a pathway contributed to the therapeutic role of 1, 25 (OH) _2_D_3_ on diabetes-induced hepatic steatosis. Moreover, we suggest that 1, 25 (OH) _2_D_3_ may play as PPAR-α agonists that could decrease plasma TG levels to modulate lipid metabolism. Nevertheless, the detailed mechanism behind PPAR-α up-regulation by 1, 25 (OH) _2_D_3_ considered as complex and remains unclear.

Recent studies have suggested that PPAR-α down-regulation may facilitate the activity of hepatic pro-inflammatory cytokines [47]. Devchand *et al*. [48] have demonstrated that PPAR-α plays a role in acute inflammation control by using the PPAR-α-deficient mice. Thus, the PPAR-α signaling pathway may become a potentially interesting target for anti-inflammatory drug development. Furthermore, several studies have shown that inflammation regulates expression of PPAR isoforms, transcriptional interference between PPAR-α and NF-κ B occurs reciprocally, since NF-κ B can inhibit PPAR-α-mediated activation of a PPAR response element-driven promoter [49]. In this study, we have demonstrated that 1, 25 (OH) _2_D_3_ can down-regulates the expression of the NF-κ B up-regulates the expression of PPAR-α in liver, and the expression of NF-κ B and PPAR-α were negatively correlated. Concordantly with a previous report [47-49], we speculate that further investigation of the molecular interaction between NF-κ B and PPAR-α in liver may help to develop therapeutic approaches for the treatment of hepatic diseases associated with diabetes mellitus.

In summary, our data provided some evidences that 1, 25 (OH) _2_D_3_ can inhibit NF-κ B and its downstream inflammatory cytokines expression and promote PPAR-α and CPT-1 expression in liver of diabetic rat, which may partly contribute to its protective effects for diabetic liver injury. Further studies are required to identify how 1, 25 (OH) _2_D_3_ can regulate the gene expression involved in hepatic proinflammatory cytokines and lipid metabolism. The relationship between vitamin D response element and the direct impact of 1, 25 (OH) _2_D_3_ on NF-κ B and PPAR-α gene expression in liver may need further investigation.

## Conclusion

This study links the effects of the 1, 25 (OH) _2_D_3_ in regulating proinflammatory cytokines and lipid metabolism in a DM liver model. 1, 25 (OH) _2_D_3_ significantly changed liver functional characteristics and fatty acid regulation, which may have been caused by its effects on hepatic PPAR-α and proinflammatory cytokines.
